# Cloning and endogenous expression of a *Eucalyptus grandis* UDP-glucose dehydrogenase cDNA

**DOI:** 10.1590/S1415-47572010005000078

**Published:** 2010-12-01

**Authors:** Mônica T. Veneziano Labate, Ana L. Ferreira Bertolo, Daniela Defávari do Nascimento, Gunta Gutmanis, Alexander de Andrade, Maria J. Calderan Rodrigues, Eduardo L. O. Camargo, Luis Felipe Boaretto, David H. Moon, Juliano Bragatto, Carlos A. Labate

**Affiliations:** Laboratório Max Feffer de Genética de Plantas, Departamento de Genética, Escola Superior de Agricultura Luiz de Queiroz, Universidade de São Paulo, Piracicaba, SPBrazil

**Keywords:** UDP-glucose, UDP-glucuronate, hemicellulose, pectin, cell wall

## Abstract

UDP-glucose dehydrogenase (UGDH) catalyzes the oxidation of UDP-glucose (UDP-Glc) to UDP-glucuronate (UDP-GlcA), a key sugar nucleotide involved in the biosynthesis of plant cell wall polysaccharides. A full-length cDNA fragment coding for UGDH was cloned from the cambial region of 6-month-old *E. grandis* saplings by RT-PCR. The 1443-bp-ORF encodes a protein of 480 amino acids with a predicted molecular weight of 53 kDa. The recombinant protein expressed in *Escherichia coli* catalyzed the conversion of UDP-Glc to UDP-GlcA, confirming that the cloned cDNA encodes UGDH. The deduced amino acid sequence of the cDNA showed a high degree of identity with UGDH from several plant species. The Southern blot assay indicated that more than one copy of *UGDH* is present in *Eucalyptus*. These results were also confirmed by the proteomic analysis of the cambial region of 3- and 22-year-old *E. grandis* trees by 2-DE and LC-MS/MS*,* showing that at least two isoforms are present. The cloned gene is mainly expressed in roots, stem and bark of 6-month-old saplings, with a lower expression in leaves. High expression levels were also observed in the cambial region of 3- and 22-year-old trees. The results described in this paper provide a further view of the hemicellulose biosynthesis during wood formation in *E. grandis*.

## Introduction

The biosynthesis of hemicelluloses and pectins in higher plants is mainly regulated by the cytosolic enzyme UDP-glucose dehydrogenase (UGDH) (EC 1.1.1.22), that catalyzes the sugar interconversion involving the four-electron, NAD^+^-linked, oxidation of UDP-glucose to UDP-glucuronate (Amino *et al.*, 1985; Gibeaut, 2000). The reaction is essentially irreversible, resulting in a unidirectional flow of UDP-glucuronate (UDP-GlcA) into a pool of sugar nucleotides, predominantly used for the biosynthesis of plant cell wall polysaccharides. UDP-GlcA is the precursor of many sugar nucleotides, including UDP-galacturonic acid (UDP-GalA), UDP-xylose (UDP-Xyl), UDP-arabinose (UDP-Ara) and UDP-apiose (UDP-Api), which are the substrates for the polymer synthases involved in the formation of pectins and hemicelluloses (Gibeaut and Carpita, 1994). UDP-glucuronate is the dominant sugar nucleotide precursor in the biosynthesis of hemicelluloses and pectins, providing half of the biomass of the primary cell wall (Zablackis *et al.*, 1995; Reiter and Vanzin, 2001; Seifert, 2004).

The presence of isoforms for UGDHs was ignored in previous studies (Tenhaken and Thulke, 1996; Stewart and Copeland, 1998; Seitz *et al.*, 2000). In *Arabidopsis*, the *UGDH* gene family is represented by four highly similar *UGDH* isoforms: *UGDH1,* earlier described by Seitz *et al.* (2000), and three others (*UGDH2-4*), located on distinct chromosomes (Reiter and Vanzin, 2001). More recently, a fifth *UGDH* pseudogene (partial sequence) with a weaker similarity was detected (Klinghammer and Tenhaken, 2007). Moreover, in poplar, at least two isoforms were reported (Johansson *et al.*, 2002). A developmentally regulated and often transient expression pattern for *UGDH* transcripts was observed by Seitz *et al.* (2000), suggesting that the isoforms are expressed in cells only when sugar nucleotides derived from the UDP-GlcA are needed for the synthesis of new cell wall polymers. Developmental regulation was also suggested by Carvalho *et al.* (2008) in *E. grandis* wood-forming tissue, where one UGDH isoform is preferentially expressed.

The biosynthesis of UDP-GlcA in plants can also occur by the inositol oxygenation pathway. In young seedlings, this alternative pathway operates and oxidizes inositol directly to GlcA, as shown by isotope-labeling experiments (Loewus *et al.*, 1973; Roberts and Loewus, 1973; Sasaki and Taylor, 1984). The first irreversible step in the oxidation of myo-inositol is catalyzed by myo-inositol oxygenase (EC 1.13.99.1), generating D-glucuronate that is phosphorylated by D-glucuronokinase (EC 2.7.1.43) to D-glucuronate 1-P (Biswas *et al.*, 1984), and immediately transformed into UDP-glucuronate by UDP-glucuronate-1-phosphate uridylyltransferase (EC 2.7.7.44). Studies on enzymes participating in the sugar nucleotide interconversion showed that UGDH is often the least active enzyme of the pathway, and is also present in low amounts, leading to the conclusion that this enzyme might be rate-limiting for the synthesis of cell wall precursors, since there is a demand for UDP-glucuronate in all plant tissues and organs at all stages of development (Dalessandro and Northcote, 1977a,b; Robertson *et al.*, 1995; Gibeaut, 2000).

In wood-forming tissues, the role of UGDH in the regulation of hemicellulose biosynthesis is still poorly understood, particularly in fast growing trees such as *Eucalyptus spp*. In the present study, we cloned a UGDH-encoding cDNA from the cambial zone of 6-month-old *E. grandis* saplings and analyzed the expression pattern of transcripts from different developing tissues and plant ages by semi-quantitative RT-PCR. The cDNA sequences for *UGDH* from woody species have previously been documented for poplar (Johansson *et al.*, 2002) and cinnamon (NCBI, AY496079), and recently for *Eucalyptus gunnii*, although incomplete (EMBL, CT982226). Therefore, this is the first complete sequence of a *UGDH* cDNA reported for *E. grandis*. Moreover, this work led to the conclusion, based on Southern blot analysis, that there are at least 2 copies of the *UGDH* gene in *E. grandis*. We also isolated and identified three UGDH protein isoforms from the cambial region of 3- and 22-year-old trees, using 2-DE gels and LC-MS/MS.

## Material and Methods

###  Plant material

Six-month-old saplings from a commercial clone of *E. grandis*, kindly provided by Suzano Papel e Celulose, were maintained in growth chambers under controlled conditions: 500 mol m^-2^ s^-1^ irradiance, 16/8 h light/dark photoperiod, and a 24 °C day/18 °C night temperature regime. The cambial region of 3- and 22-year-old-trees, containing the differentiating xylem and phloem tissues, was sampled by removing the bark and scrapping the inner parts of the stems with a razor blade, and immediately freezing it in liquid nitrogen, as described by Celedon *et al.* (2007).

###  Design of degenerate primers

**Figure 1:**
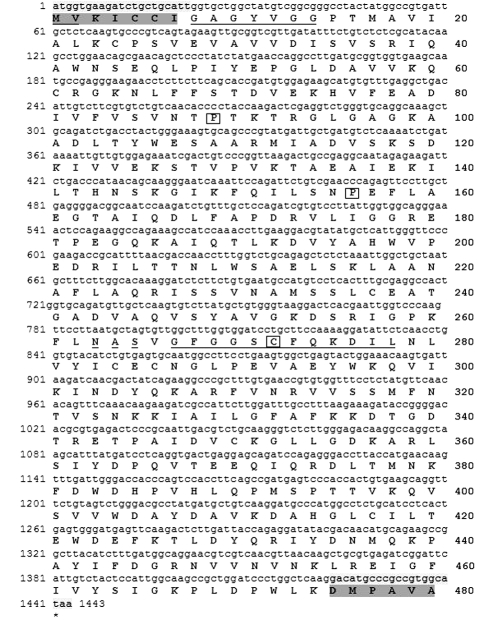
Nucleotide (nt) and derived amino acid (aa) sequence of the cloned cDNA of the *E. grandis UGDH* gene. Underlined are the translation initiation nt motif (aa# 1 and 2), the NAD cofactor binding site (aa# 8-14) and the catalytic site (aa# 267-278, with a Cys residue, boxed). A putative glycosylation site (aa# 263-265) is underlined with a broken line. Pro residues at 89 and 156 are also boxed. The conserved N and C terminal regions (highlighted in dark grey) were used for the alignment of the 21 nucleotides from the 5'and 3'-ends (highlighted in light grey) of the cloned cDNA against poplar (AF053973 and AY466400), soybean (U53418), *Arabidopsis* (AP001309), taro (AY222335), cinnamon (AY496079), rice (AK103919), wheat (BT009444) and maize (AY103689), to produce the degenerate primers. The complete nt-sequence of *E. grandisUGDH* was deposited in the NCBI GenBank under accession nº EF179384.

The UGDH-encoding cDNA of *E. grandis* was cloned, using the RT-PCR technique employing degenerate primers to highly conserved 5' and 3'ends (Kunihiro *et al.*, 2002). To accomplish this, a BLAST search (Altschul *et al.*, 1977) was performed for plant orthologous sequences encoding UGDH protein. A preliminary analysis of the highly conserved N and C terminal regions among amino acid sequences of UGDH was made by means of the ClustalW2 program (Thompson *et al.*, 1994), using soybean (AAB58398), *Arabidopsis* (BAB02581), rice (XP468764), cinnamon (AAR84297), poplar (AAF04455 and AAR32717), and taro (AA062313). The corresponding nucleotide sequences from soybean (U53418), *Arabidopsis* (AP001309), cinnamon (AY496079), poplar (AF0539973 and AY466400), taro (AY222335), rice (AK103919), wheat (BT009444) and maize (AY103689) were used to construct the degenerate primers flanking the *E. grandisUGDH* ORF. The forward (5'ATGGTGAAGAT**H**TG**Y**T G**Y**AT**Y**3') and reverse (5'TTA**D**GC**V**A**YV**GC**R**GGCA TGTC3') primers were forced to flank the complete *UGDH* open reading frame. The alignment of the 21 nucleotides from the 5'and 3' ends of the nine sequences showed that the differences among them were mainly in the third base of the codon, not changing the amino acid, with few exceptions (Figure 1). These minor differences were overcome by the use of degenerate primers. Primer sequences are represented in standard IUB/IUPAC amino acid and nucleic acid codes.

###  Cloning and sequence analysis of the *UGDH* cDNA

Total RNA was isolated from the cambial region of 6-month-old saplings of *E. grandis* using the method of Salzman *et al.* (1999), and poly(A) mRNA was purified from 75 μg of total RNA, using the Dynabeads mRNA Purification kit (Dynal) as specified by the manufacturer, and eluted in 20 μL Tris-HCl 10 mM.

A UGDH encoding cDNA was obtained by RT-PCR, using the SuperScript One-step RT-PCR with Platinum Taq (Invitrogen), and the specific flanking primers (*UGDH*_ Forward:_5'**CACC**ATGGTGAAGATHTG YTGYATY3';_*UGDH_*Reverse: 5'TTADGCVAYVGCR GGCATGTC3') for the corresponding open reading frame. The directional sequence CACC was added to the sense primer to facilitate the cloning orientation of the cDNA. For cDNA synthesis, a 50 μL RT-PCR reaction containing 25 μL reaction mix (0.4 mM of each dNTP, 2.4 mM of MgSO_4_), 100 pg of mRNA, 0.2 μM of each primer, and 1 μL of RT/Platinum *Taq* mix was prepared. The cDNA was synthesized at 50 °C for 30 min and denatured at 94 °C for 2 min, and amplification was performed using 38 cycles of 15 s at 94 °C, 30 s at 60 °C, 1 min and 30 s at 72 °C, followed by 7 min at 72 °C. The 1443-bp blunt-end amplification product was cloned into the pENTR-Directional-TOPO^®^ Cloning Vector, for entry into the Gateway System (Invitrogen), according to the manufacturer's instructions. A *UGDH* recombinant clone, selected in kanamycin (50 μg mL^-1^), was purified and screened by PCR for the presence of full-length *UGDH* cDNA, using degenerate primers and also by sequencing in both directions with the universal M13 primers and theBigDye Terminator Cycle sequencing kit (Perkin Elmer), to ensure the proper reading frame. The samples were loaded onto an ABI 3100 sequencer (Perkin Elmer). The complete nt sequence of the *E. grandisUGDH* was deposited in the NCBI GenBank under accession nº EF179384.

###  Expression of UGDH protein in *E. coli*

The RT-PCR product corresponding to the *E. grandisUGDH* cDNA was sub-cloned into the Gateway^®^pDEST17 vector (Invitrogen) in frame with a N-terminal 6x His tag, for expression of the recombinant protein, using the *E. coli* Expression System with Gateway^®^ technology.

For the protein expression, the *UGDH* recombinant pDEST17 vector was introduced into chemically competent *E. coli* BL21-AI (Invitrogen) by heat shock. A recombinant clone, previously screened by PCR for the presence of the *UGDH* insert, was grown in LB medium containing ampicillin (100 μg mL^-1^) at 37 °C and 200 rpm. This starter culture (1 mL, OD_600nm_ = 0.7) was used to inoculate 50 mL of LB medium supplemented with ampicillin (100 μg mL^-1^) and left to grow at 37 °C (200 rpm) until an OD_600nm_ of 0.4 was reached. After 3 h of induction with 0.2% (w/v) L-arabinose, the bacterial cells were harvested by centrifugation at 13,000 *g* for 1 min. The pellet was resuspended in 5 mL of lysis buffer (100 mM NaH_2_PO_4_, pH 8.0; 10 mM Tris, containing 0.1% (w/v) lysozyme, 1 mM PMSF, 10 mM ß-mercaptoethanol, 10 μg RNAseA mL^-1^, 5 μg DNAse mL^-1^) and sonicated (three times of 10-second bursts at medium intensity). The resulting lysate was separated into soluble and insoluble fractions by centrifugation (3,000 *g*) for 15 min.

###  Purification of the recombinant protein

The histidine fusion protein was purified from the soluble fraction using a purification column prepared with ProBondNickel-Chelating Resin (Invitrogen), according to the manufacturer's instructions. The purity and *M*_r_ of the proteins in the eluted fractions collected were analyzed by 12.5% (w/v) SDS-PAGE, using Coomassie brilliant blue G-250 staining (Candiano *et al.*, 2004).

Protein concentration was determined using the Bradford assay (Bradford, 1976) and bovine serum albumin as a standard with protein dye reagent (Bio-Rad), following the supplier's instructions.

###  Enzyme assay

The activity of the purified Poly-His-Tagged eucalyptus UDP-GDH was measured at 30 °C (Stewart and Copeland, 1999) in a continuous assay, by monitoring, with a Hitachi U3300 spectrophotometer, the increase in absorbance at 340 nm due to the UDP-Glc-dependent formation of NADH. Reaction mixtures for the standard assay contained, in a final volume of 1 mL, 20 mM Tris-HCL, pH = 8.0, and 2 mM MgCl_2_. The kinetic data for NAD^+^ or UDP-Glc as substrates were determined in triplicate assays, keeping one of the substrates at saturating concentrations. The assay was initiated by the addition of UDP-Glc, and activity was calculated from linear initial reaction rates, based on the assumption that 2 mol of NADH were formed per mole of oxidized UDP-Glc.

###  Protein extraction for the two-dimensional gel electrophoresis

Total proteins from the cambial region of 3- and 22-year-old *E. grandis* trees were extracted as described by Hurkman and Tanaka (1986), with few modifications introduced by Celedon *et al.* (2007).

###  Two-dimensional gel electrophoresis

Protein samples (containing 2 μg of protein μL^-1^) were applied onto a 4-7 linear immobilized pH gradient strip (18 cm, GE HealthCare) and focalized using the IPGphor apparatus (GE HealthCare). Strips were rehydrated for 12 h at 20 °C and 50 V. The proteins were prefocused at 100 V for 1 h and then at 200 V for 1 h, 400 V for 1 h , 700 V for 1 h, 1000 V for 1 h, and finally focused for a total of 70 KVh. After isoeletric focusing (IEF), the strips were kept at -80 °C until needed.

For the second-dimension analysis, the strips were kept at room temperature for 15 min in equilibration buffer [6 M urea, 2% (w/v) SDS, 50 mM Tris-HCl pH 6.8, 30% (v/v) glycerol] containing 1% (w/v) DTT, followed by incubation in the same buffer added with 2.5% (w/v) iodoacetamide and 0.001% bromophenol blue. The second-dimension electrophoresis was performed in 12% (w/v) polyacrylamide gels at 30 mA, until the dye reached the bottom of the gel. Three replicates were performed for each sample. Proteins were detected using Coomassie Brilliant Blue G-250 (Candiano *et al.*, 2004), with modifications. Gels were incubated twice for 1 h each in a solution containing 3% (v/v) phosphoric acid and 50% (v/v) ethanol, and once for 1 h in 2% (v/v) phosphoric acid. Protein detection was done after leaving the gels overnight in staining solution [17% (v/v) methanol, 15% (w/v) ammonium sulfate, 2% (v/v) phosphoric acid plus 0.1% (w/v) dye], followed by 3 washes in water (10 min each). The gels were then stored in 15% (w/v) ammonium sulfate for image analysis and spot selection.

###  In-gel protein digestion

Protein spots were excised from SDS-PAGE gels, cut into 1 mm cubes and washed with water for 15 min. For destaining, the gel pieces were washed several times with a 50% (v/v) acetonitrile (ACN) solution containing 50 mM ammonium bicarbonate, until complete removal of the Coomassie (G250) stain. The 2-DE gel spots were completely dehydrated with 100% (v/v) ACN for 10 min, rehydrated with 50 mM ammonium bicarbonate plus 20 mM dithiothreitol (DTT), and maintained for 40 min at 60 °C. This solution was discarded and replaced by 50 mM ammonium bicarbonate with 55 mM iodoacetamide, before keeping the tubes in darkness for 30 min. Then the gel pieces were dehydrated again with 100% (v/v) ACN and let to air-dry for complete removal of the solvent. Protein digestion was carried out with a solution of 10 ng μL^-^1 trypsin (Promega) in 25 mM ammonium bicarbonate, for 12 h at 37 °C. Gel pieces were extracted twice with 50 μL of 60% (v/v) ACN containing 1% (v/v) formic acid (FA), and once with 50 μL of 100% ACN. All supernatants were combined and vacuum-dried. The peptides were then resuspended in 12 μL 1% (v/v) FA for MS analysis.

###  Protein identification by LC-MS/MS

The peptides were separated and identified by on-line chromatography, using a Cap-LC coupled to a Q-TOF Ultima API mass spectrometer (Waters, UK). 5 mL of sample were loaded onto a 0.18 mm x 23.5 mm NanoEase Trapping Column (Waters, UK) for pre-concentration and desalination, followed by peptide separation on a C18, 3.5 μm, 75 μm x 100 mm NanoEase Symmetry 300 LC column (Waters, UK). Peptides were eluted in a 60 min linear gradient of solvent B [95% (v/v) ACN containing 0.1% (v/v) formic acid in water] and solvent A [5% (v/v) ACN containing 0.1% (v/v) formic acid in water], at a flow rate of 250 nL min^-1^.

###  MS spectra analysis

All analyses were performed using a positive ion mode at a 3 kV needle voltage. The mass range was set from 300 to 2000 m/z, and the MS/MS spectra acquired for the most intense peaks (≥ 15 counts). Multiple charged precursor ions were selected for fragmentation and peptide sequencing, using automated data-dependent-acquisition (DDA) MassLynx software (Waters), switching from the MS to the MS/MS mode and then returning to MS. The resulting fragmented spectra were processed using the ProteinLynx v. 4.0 software (Waters), and the MASCOT MS/MS Ion Search was used to blast the sequences against the SwissProt and NCBI databank. Combined MS-MS/MS searches were conducted with parent ion mass tolerance at 50 ppm, MS/MS mass tolerance of 0.1 Da, carbamidomethylation of cysteine (fixed modification) and methionine oxidation (variable modification). According to MASCOT probability analysis, only significant hits (p < 0.05) were accepted.

###  Southern blot analysis

Genomic DNA (15 μg) was isolated from *Eucalyptus grandis* leaves, as described by Doyle and Doyle (1987), and digested with *Hin*dIII, *Eco*RI, *Bam*HI, *Eco*RV, *Nco*I and *Sac*I restriction enzymes at 37 °C for 16 h. The DNA fragments were separated on 1% (w/v) agarose gel in 0.5 x TBE buffer (45 mM Tris-borate, 1 mM EDTA, pH 8.0), stained with ethidium bromide, visualized under ultraviolet light, and denaturated before being transferred onto a Hybond N^+^ nylon membrane (GE HealthCare) by capillary transfer (Southern, 1975; Sambrook *et al.*, 1989). The membrane was probed using the AlkPhos Direct Labeling Module (GE HealthCare). The alkaline-phosphatase-labeled-PCR product (698-bp) resulting from the amplification of a conserved internal region (nt# 188-886, Figure 1) of the eucalyptus cDNA (1443-bp) cloned with the following primers (*ugdhDForward:* 5'-GAAGAACCTCTT CTTCAGCA-3' *ugdhDReverse:* 5'-AGTACTCAGCCAC TTCAGGA-3') synthesized for the UGDH domain (pfam03721, pfam00984, pfam03720), used as probe, was detected with the Gene Images CDP-Star Detection Module (GE HealthCare). Hybridization was carried out at 60 °C for 16 h, followed by stringent washes: twice in fresh primary wash buffer [2 M urea, 50 mM NaH_2_PO_4_, pH 7.0, 150 mM NaCl, 1 mM MgCl_2_ containing 0.1% (w/v) SDS and 0.2% (w/v) blocking reagent] at 55 °C for 10 min, and once in secondary wash buffer [50 mM Tris, 100 mM NaCl containing 2 mM MgCl_2_] at room temperature for 5 min. The hybridization signal was recorded on X-ray film (MXG/PLUS, Kodak) after 1 h exposure between intensifying screens (GE HealthCare), at room temperature.

The resulting exposed X-ray film was used as reference for localization of the bands.

###  Semi-quantitative real-time PCR (RT-PCR)

Tissue samples from 6-month-old saplings and from 3- and 22-year-old plants were used for total RNA isolation, as described by Salzman *et al.* (1999). Poly(A) mRNAs were purified from 75 μg of total RNA, using the Dynabeads mRNA Purification kit (Dynal) as specified by the manufacturer, and eluted in 20 μL Tris-HCl 10 mM. First-strand cDNAs were generated using the SuperScript III First-Strand Synthesis SuperMix in a 20 μL reaction mixture, containing 3/10 of the eluted mRNA, 50 ng random hexamers, and 1 μL annealing buffer. After 5 min of incubation at 65 °C, 10 μL of 2x First-Strand Reaction Mix plus 2 μL SuperScript III/RNase OUT Enzyme Mix, which includes the SuperScript III Reverse Transcriptase, were added, and the final reaction mixture was incubated for 10 min at 25 °C, followed by 50 min at 50 °C, and 5 min at 85 °C, following the manufacturer's instructions (Invitrogen). Semi-quantitative RT-PCR assays were performed, using 1/10 of the cDNA preparation per PCR, based on a preliminary semi-quantitative RT-PCR (data not shown). Primer sequences and amplicon sizes were the following: *UGDH* (247-bp; primers designed specifically for the cloned eucalyptus cDNA forward 5'- GCCCGTATGATTGCTGATGTC -3', reverse 5'- TCAAGGTTTGGATGGCTTTC-3'); *ubiquitin* (223-bp, primers to the *E. grandis* constitutive ubiquitin gene, forward 5'-CGATTGATTCTCAGCAAGC-3', reverse 5'- GGATGTTGTAGTCAGCCAAGG-3'). Amplification specificity was checked by melting-curve analysis, and PCR efficiency determined using standard curves for each primer pair constructed with serial dilutions (1:10, 1:100 and 1:1000) of the cDNA preparation.

## Results and Discussion

###  PCR amplification of the full-length cDNA encoding *E. grandis* UGDH

In this study, the degenerate primers designed to anneal highly conserved 5' and 3' regions of already known genes, in combination with the RT-PCR technique, proved to be a potential strategy to overcome the difficulties in cloning a particular novel gene, when cDNA or a genomic library are not available. The RT-PCR reaction produced a 1443-nt-long cDNA sequence, with an open reading frame of 480 amino acids encoding UGDH (Figure 1).

###  Nucleotide and deduced amino acid sequence analysis

The deduced amino acid sequence for eucalyptus shows conserved motifs (pfam03721, pfam00984, pfam03720). Figure 1 (underlined) presents the translation initiation nt motif (aa# 1 and 2), the NAD cofactor binding site (aa# 8-14), the catalytic site (aa# 267-278, with a Cys residue, boxed). A putative glycosylation site (aa# 263-265), also found in poplar (Johansson *et al.*, 2002), is indicated with a broken line, and Pro residues at aa# 89 and 156 are also boxed, representing the main chain bends in the protein structure (Hempel *et al.*, 1994). The derived amino acid sequence from the database search BLASTX analysis (Altschul *et al.*, 1997) showed a high degree of identity to the UGDH of soybean (92%), cinnamon (90%), *Arabidopsis* (90%), taro (89%), poplar (89%), and rice (85%), among several plant species, including some putatives UDP-glucose dehydrogenases, such as that of tobacco (91%). The high identity among UGDHs of higher plants suggests strict structural requirements for proper functioning of the protein. It is interesting to observe the high degree of amino acid sequence identity of the eucalyptus UGDH, even along the N and C terminal regions (Figure 1, highlighted in dark grey), where the differences in the first 25 aa, were less than 4% and 16%, respectively, compared to the leguminosa soybean and gramineae such as rice, maize and, particularly, wheat. The analysis of the phylogenetic tree, generated using the CLC Main Work Bench 5.5 (UPGMA algorithm with Bootstrap analysis, 100 replicates) for the eucalyptus UGDH protein sequence and eight other plant species, shows a high similarity of the *E. grandis* protein sequence to various plant sequences, particularly soybean and *Arabidopsis* (Figure S1, supplementary data).

###  Expression of recombinant eucalyptus UGDH

The recombinant UGDH has been found to be often present in protein inclusion bodies (Tenhaken and Thulke, 1996; Hinterberg *et al.*, 2002). It is known that eukaryotic proteins expressed in *E. coli* often form protein inclusion bodies, due to differences in the protein-folding systems between prokaryotes and eukaryotes (Oka and Jigami, 2006). Therefore, polyhistidine recombinant eucalyptus UGDH protein was produced in BL21-AI cells, and purified under a narrow range of optimized conditions, which included a low centrifugation of the lysate to recover, under native conditions, enough enzymatically active protein in the soluble fraction. The protein was purified at 4 °C, and immediately used for the enzyme assay.

**Figure 2:**
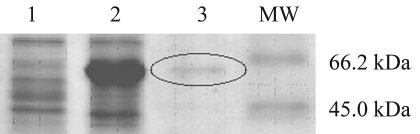
Coomassie-stained SDS-PAGE of recombinant UGDH protein. The eucalyptus *UGDH* cDNA cloned into a His-tagged pDEST expression vector was used to transform the BL21 *E. coli* cells. (1) Extracts from uninduced cells; (2) L-arabinose-induced cells; and (3) the purified recombinant eucalyptus UGDH from the L-arabinose-induced *E. coli* culture. MW: Molecular weight marker (Sigma).

The Coomassie-stained SDS-PAGE gel illustrates the L-arabinose induction of the UGDH recombinant protein in *E. coli* (Figure 2). The identification of the protein was confirmed by LC-MS/MS, and the peptides sequenced covered 23.33% of the total protein, with an estimated molecular weight of 52.91 kDa, and a 100% probability that the peptides are related to the expected protein (Table S1, supplementary data).

**Figure 3:**
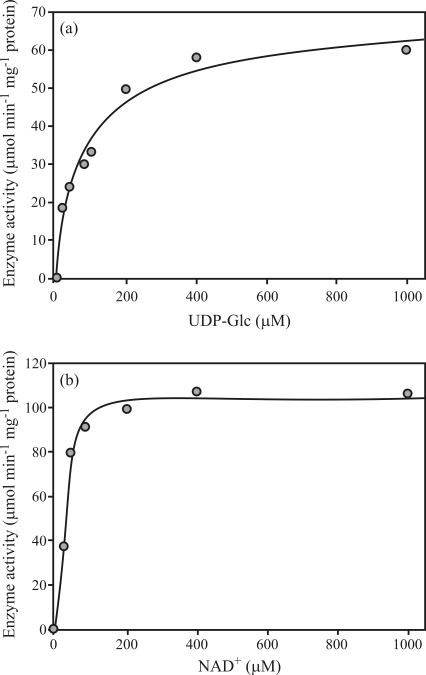
Kinetic data of the eucalyptus UDP-GlucDH. The steady-state enzyme activity was measured as conversion of NAD^+^ to NADH detected by the absorbance at 340 nm. Purified eucalyptus UDP-GlucDH was incubated for 1 min with increasing concentrations of NAD^+^ (0-1 mM) in the presence of saturating substrate (A, UDP-Gluc, 1 mM), or with increasing concentrations of UDP-Gluc (0-1 mM) in the presence of saturating cofactor (B, NAD^+^, 1 mM).

The activity of the purified Poly-His-Tagged-UGDH measured at 30 °C (Stewart and Copeland, 1999), in a continuous assay, by spectrophotometrically monitoring the absorbance increase at 340 nm due to the UDP-Glc-dependent formation of NADH, showed that the recombinant protein expressed in *E. coli* catalyzed the conversion of UDP-Glc to UDP-GlcA (Figure 3), thus confirming that the cloned cDNA encodes the UGDH enzyme.

###  Enzyme kinetics

The recombinant protein expressed in *E. coli* catalyzed the conversion of UDP-Glc to UDP-GlcA, confirming that the cloned cDNA encodes the UGDH enzyme (Figure 3). The kinetic data were then fitted to the Michaelis-Menten equation to obtain the *K*_m_ and *V*_max_ values (Segel, 1976). For both substrates, UDP-Glc and NAD^+^, hyperbolic saturation curves were observed and the *K*_m_ values calculated, using the Origin-software^®^, 60.7 ± 8.5 μM for UDP-Glc (*V*_max_ = 67.9 ± 9.2 μmol min^-1^ mg protein^-1^) and 67.3 ± 17.9 μM for NAD^+^ (*V*_max_ = 171.8 ± 34.8 μmol min^-1^ mg protein^-1^) (Figures 3A and 3B, respectively). The estimated *K*_m_ of the eucalyptus enzyme for NAD^+^ was similar to the value described for the recombinant protein from soybean (70 ± 5 μM), whereas the *K*_m_ for UDP-Glc was around 3 times higher (22 ± 2 μM) (Hinterberg *et al.*, 2002). A characterization of three isoforms of UGDH (UGDH2, UGDH3 and UGDH4) from *Arabidopsis* evidenced a similar affinity value for the cofactor NAD^+^ as that observed for eucalyptus (40-45 μM), while major differences were observed in the *K*_m_ for UDP-Glc: 123 ± 9 μM, 335 ± 16 μM and 171 ± 9 μM, respectively (Klinghammer and Tenhaken, 2007). The high affinity of the enzyme for NAD^+^ was also observed in eucalyptus, confirming previous observations that the UGDHs are not limited by NAD^+^ levels *in vivo*, while UDP-Glc affinity might be isoform-dependent (Hinterberg *et al.*, 2002; Klinghammer and Tenhaken, 2007).

###  Genomic copies of *UGDH*

**Figure 4:**
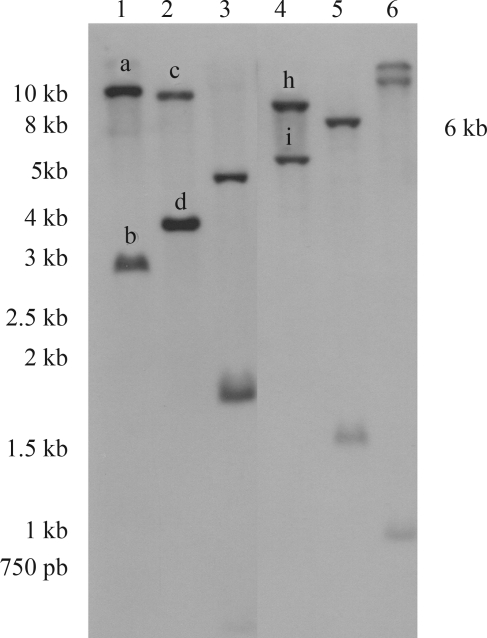
Genomic Southern blot of *E. grandis* DNA, restricted with *Hin*dIII (1), *Eco*RI (2), *Bam*HI (3), *Eco*RV (4), *Nco*I (5), and *Sac*I (6). The sizes of the fragments are indicated in kilobase pairs (kb). The alkaline-phosphatase-labeled PCR product (698-bp) resulting from the amplification of a conserved internal region (nt# 188-886, Figure 1) of the cloned *E. grandis* cDNA (1443-bp) was used as probe.

Southern blot analysis indicated the existence of a least two copies of the *UGDH* gene within the eucalyptus genome (Figure 4). The existence of a single-copy *UGDH* gene has been described for soybean (Tenhaken and Thulke, 1996) and *Arabidopsis* (Tenhaken and Thulke, 1996; Seitz *et al.*, 2000). In poplar (Johansson *et al.*, 2002), two highly homologous genes were found, as also observed in maize (Kärkönen *et al.* 2005). However, a later evaluation of the *Arabidopsis* and poplar genome projects indicated multiple copies. The presence of four UGDH isoforms in *Arabidopsis*, highly similar to each other, reported by Reiter and Vanzin (2001), was confirmed by Klinghammer and Tenhaken (2007), who found a fifth *UGDH* pseudogene, located on chromosome III, with a weaker similarity to the others. In the *Populus trichocarpa* genome (*Populus* genome project), four *UGDH* genes were identified, one in linkage group IV (224080232), one in linkage group VIII (224098951), one in linkage group X (224112137), and one in linkage group XVII (224141486).

**Figure 5:**
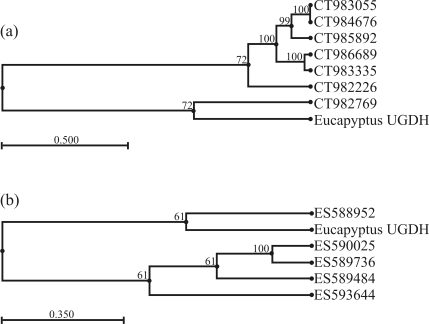
Phylogenetic tree generated using CLC Main Work Bench 5.5 (UPGMA algorithm with Bootstrap analysis, 100 replicates) for the complete nucleotide sequence of the cloned *E. grandisUGDH* mRNA (144926038): A) using seven *E. gunnii* EST sequences (103476385, 103475842, 103479156, 103478292, 103476672, 103476952, 103480206) of mRNA isolated from differentiating xylem; B) using five *E. globulus* EST sequences (162324959, 162326130, 162329222, 162329166, 162326954) of mRNA isolated under low temperature conditions from leaf tissue.

We further analyzed the possible number of copies for *UGDH* in the *Eucalyptus* genome by performing a tblastx analysis of the eucalyptus ESTs sequences available in the GenBank database. The results showed that the cloned *UGDH* sequence from *E. grandis* is highly similar (~99%) to the amino acid sequences of seven ESTs produced from the differentiating xylem of *Eucalyptus gunnii* (103476385, 103475842, 103479156, 103478292, 103476672, 103476952, 103480206) and five *Eucalyptus globulus* EST sequences, using mRNA isolated from leaf tissue under low temperature conditions (162324959, 162326130, 162329222, 162329166, 162326954). The phylogenetic tree generated using CLC Main Work Bench 5.5 (UPGMA algorithm with Bootstrap analysis, 100 replicates) for the complete nucleotide sequence of the cloned *E. grandis* UGDH mRNA (144926038) with these seven cambial *E. gunnii* EST and the five leaf *E. globulus* sequences (Figures 5A and 5B, respectively), suggests that there are at least two groups of nucleotide sequences, most probably indicating two isoforms of this gene in the RNA extracted from the cambial and leaf tissues.

**Figure 6:**
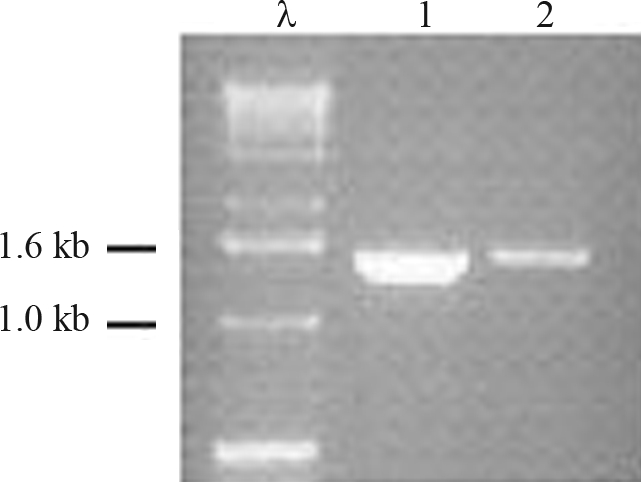
Amplification product (1443-bp) of the eucalyptus genomic DNA (1), and the corresponding cDNA (2) for eucalyptus *UGDH*, against the degenerate primers flanking the *UGDH* open reading frame. λ: 1 kb DNA ladder (Invitrogen), kb: kilobase pair.

The amplification product obtained from the eucalyptus genomic DNA against the primers flanking the *UGDH* open reading frame, corresponds exactly to the same size (1443-bp) of the eucalyptus *UGDH* cDNA (Figure 6), showing the lack of introns along the cloned *UGDH* gene sequence. The restriction map of the *UGDH* ORF (Figure S2, supplementary data) shows that the restriction enzymes *Eco*RI, *Hin*dIII and *Eco*RV do not cut the cloned fragment internally. This indicates that the bands observed in the Southern blot assay (Figure 4, lanes 1, 2 and 4), which are all larger than 1.6 kb, most probably contain the complete cloned *UGDH* ORF.

**Figure 7:**
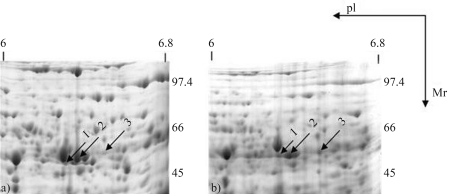
2-DE gels from the cambial region of 3-year-old (A) and 22-year-old (B) *E. grandis* trees. Arrows indicate the spot numbers of UGDH isoforms. Ip: Isoeletric-point, *M*_r:_ Molecular weight in kiloDaltons (kDa).

Moreover, in order to investigate the existence of UGDH protein isoforms, we produced 2DE-PAGE gels with proteins isolated from the cambial region of 3- and 22-year-old *E. grandis* trees. Figure 7 shows the identification of three distinct spots (1, 2 and 3) with the peptide data used to identify the proteins shown in Table S2 (supplementary data). Spot 3 showed a *M*_r_ of ~53.0 kDa and a pI of approximately 6.5, which is in accordance with the calculated values for the *M*_r_ and pI of the cloned sequence (53.09 kDa and 6.52, respectively). These values are similar to those obtained for soybean (52.94 kDa and 5.81) and poplar (52.99 kDa and 6.20). Thus, spots 1 and 2 represent proteins with different characteristics (lower pI and lower *M*_r_), indicating the existence of at least one more copy of *UGDH* in the *E. grandis* genome. The differences between spots 1 and 2 might be explained by posttranslational modification, such as phosphorylation, although further analysis is needed to confirm this hypothesis.

### *UGDH* expression in different tissues of 6-month-old saplings and in the cambial region of juvenile and mature *E. grandis* wood

**Figure 8:**
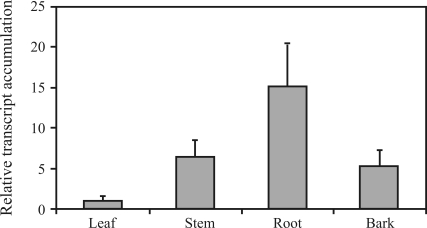
Semi**-**quantitative RT-PCR of *UGDH* transcript accumulation in roots, stem, bark and leaves of 6-month-old *E. grandis* saplings. Transcript levels were normalized relative to the ubiquitin expression level as internal standard. Results are expressed as mean of three replicates and standard deviation relative to the leaf expression level, to which the value 1 on the linear scale was assigned.

The expression of *UGDH* in different tissues of 6-month-old saplings and in the cambial region of 3- and 22-year-old trees was analyzed by semi-quantitative RT-PCR. In the 6-month-old saplings the maximum expression was observed in roots, whereas in stem and bark it was intermediate, and the leaves displayed the lowest expression (Figure 8). Similar results were observed in tobacco plants by Bindschedler *et al.* (2005), who compared the expression of *UGDH* and *NtADH2* (the dual specific *UGDH* similar to *ADH*), suggesting that the isoforms of *UGDH* might be preferentially involved in primary rather than in secondary growth, since they were strongly expressed in roots and in the youngest internodes of the stem, with the lowest level of expression observed in buds and in tobacco leaves, similar to the expression pattern observed by Seitz *et al.* (2000) in *Arabidopsis*.

**Figure 9:**
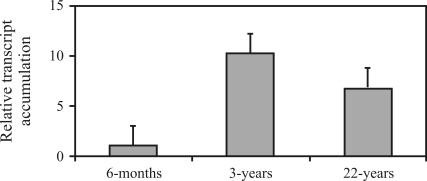
Semi-quantitative RT-PCR of *UGDH* transcript accumulation of the cambial region from 3- and 22-year-old *E. grandis* trees. Transcript levels were normalized relative to the ubiquitin expression level as internal standard. Results are expressed as mean of three replicates and standard deviation relative to the stem expression level of the 6-month-old *E. grandis* saplings, to which the value 1 on the linear scale was assigned.

We then checked the role of *UGDH* in the wood-forming tissues of juvenile and mature trees, by comparing the level of expression relative to the stem of 6-month-old saplings. Figure 9 shows that the expression of *UGDH* in the cambial region of 3- and 22-year-old trees was significantly higher than in the stem of 6-month-old saplings, indicating that the enzyme may have an important role in controlling hemicellulose biosynthesis during wood formation.

In this study, the strategy of using degenerate primers in combination with the RT-PCR technique, proved to be efficient to overcome the difficulties in cloning a particular novel gene, when cDNA or a genomic library are not available. We were able to clone and express the recombinant UGDH protein of *E. grandis* and to confirm its activity and sequence. The role of UGDH in wood-forming tissues is still poorly understood, and the isolation of *E. grandis* cDNA offers an opportunity to further understand the importance of this enzyme in trees.

## Supplementary Material

The following online material is available for this article:

Table S1 Peptide sequencing data of the recombinant UGDH protein shown in Figure 2, obtained by LC-MS/MS.

Table S2 Peptide sequencing data of UGDH proteins shown in Figure 7, identified by LC-MS/MS.

Figure S1 Phylogenetic tree generated for the eucalyptus UGDH protein sequence and eight other plant species.

Figure S2 Restriction map of the cloned *E. grandis UGDH* cDNA.

This material is available as part of the online article from http://www.scielo.br/gmb.
