# Supplementary material for: Cloning and endogenous expression of a Eucalyptus grandis UDP-glucose dehydrogenase cDNA
Source: Genet Mol Biol. 2010 Dec 1;33(4):686–95. doi: 10.1590/S1415-47572010005000078 (PMC3036151; doi:10.1590/S1415-47572010005000078)
Supplement: Table S1 — Peptide sequencing data of the recombinant UGDH protein shown in Figure 2, obtained by LC-MS/MS. [file gmb-33-4-686-suppl1.pdf]

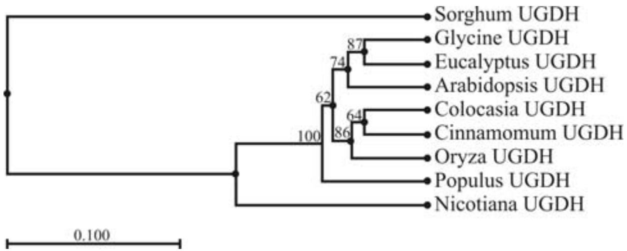

Figure S1 - Phylogenetic tree generated using CLC Main Work Bench 5.5 (UPGMA algorithm with Bootstrap analysis, 100 replicates) for the eucalyptus UGDH protein sequence and eight other plant species. The species and *gi* numbers are: *Eucalyptus grandis*, 144926039; *Cinnamomum osmophloeum*, 40317278; *Populus tomentosa*, 39939262; *Glycine Max*, 1518540; *Arabidopsis thaliana*, 11994517; *Sorghum bicolor*, 242047160; *Nicotiana tabacum*, 48093457; *Colocasia esculenta*, 29028306; *Oryza sativa* Japonica, Group 215737390.
