# Supplementary material for: Cloning and endogenous expression of a Eucalyptus grandis UDP-glucose dehydrogenase cDNA
Source: Genet Mol Biol. 2010 Dec 1;33(4):686–95. doi: 10.1590/S1415-47572010005000078 (PMC3036151; doi:10.1590/S1415-47572010005000078)
Supplement: Figure S1 — Phylogenetic tree generated for the eucalyptus UGDH protein sequence and eight other plant species. [file gmb-33-4-686-suppl3.pdf]

Table S1 - Peptide sequencing data of the recombinant UGDH protein of Figure 2, obtained by LC-MS/MS.

Name<br>EC 1.1.1.22	% Probability	Peptide matches	Coverage (%)	Mr[1)]	pI[2)]
AAB58398	100	12	23.33	52.90816	5.93

Submitted mass	Experimental mass	Peptide sequences
600.304	1198.592	(K)RAFFSTDVEK(H)
951.99	1901.964	(K)HVFEADIVFVSVSQPTK(T)
677.302	1352.588	(K)AADLTYWESAAR(M)
693.825	1385.634	(K)DVYAHWVPEDR(I)
738.401	1474.786	(R)ILTTNLWSAELSK(L)
752.393	1502.77	(R)ILTTNLWSAELSR(L)
537.79	1073.564	(K)LAANAFLAQR(I)
809.881	1617.746	(K)FLNASVGFGGSCFQK(D)
816.898	1631.78	(K)FLNASVGFGGSCFQK(D)
554.344	1106.672	(K)KIAILGFAFK(K)
731.863	1461.71	(K)KVSVVWDAFEATK(D)
740.368	1478.72	(K)KVSVVWDAYDAVK(D)

[1)] Molecular weight in kDa.
[2)] Isoelectric-point.
