# Supplementary material for: Cloning and endogenous expression of a Eucalyptus grandis UDP-glucose dehydrogenase cDNA
Source: Genet Mol Biol. 2010 Dec 1;33(4):686–95. doi: 10.1590/S1415-47572010005000078 (PMC3036151; doi:10.1590/S1415-47572010005000078)
Supplement: Figure S2 — Restriction map of the cloned E. grandis UGDH cDNA. [file gmb-33-4-686-suppl4.pdf]

Table S2 - Peptide sequencing data of UGDH proteins of Figure 7, identified by LC-MS/MS.

Spot 1

Name<br>EC 1.1.1.22	Probability (%)	Peptide matches	Coverage (%)	Theoretical [1)]Mr/pI[2)]	Experimental [1)]Mr/pI[2)]
AAR32717	100	4	9	52.963/5.92	57.907/6.39

Submitted mass	Charge	Experimental mass	Peptide sequences
490.3026	2	490.3026	(K)IAILGFAFK(K)
537.7942	2	1073.5738	(K)LAANAFLAQR(I)
677.3171	2	1352.6196	(K)AADLTYWESAAR(T)
738.3889	2	1474.7632	(R)ILTTNLWSAELSK(L)

Spot 2

Name<br>EC 1.1.1.22	Probability (%)	Peptide matches	Coverage (%)	Theoretical [1)]Mr/pI[2)]	Experimental [1)]Mr/pI[2)]
AAO62313	99.99	6	12	52.947/6.06	57.625/6.44

Submitted mass	Charge	Experimental mass	Peptide sequences
382.2243	2	762.4340	(R)MIADVSK(S)
398.2232	2	794.4318	(K)TLDYQR(I)
537.8185	2	1073.6224	(K)LAANAFLAQR(I)
677.3301	2	1352.6456	(K)AADLTYWESAAR(M)
462.9116	3	1385.7130	(K)DVYAHWVPEDR(I)
738.4020	2	1474.7894	(R)IITTNLWSAELSK(L)

Spot 3

Name<br>EC 1.1.1.22	Probability (%)	Peptide matches	Coverage (%)	Theoretical [1)]Mr/pI[2]	Experimental [1)]Mr/pI[2)]
AAR32717	99.99	3	7	53.543/5.92	59.101/6.61

Submitted mass	Charge	Experimental mass	Peptide sequences
490.3130	2	978.6114	(K)IAILGFAFK(K)
677.3294	2	1352.6442	(K)AADLTYWESAAR(T)
738.4080	2	1474.8014	(R)ILTTNLWSAELSK(L)

[1)]Molecular weight in kDa.
[2)]Isoelectric-point.
