# Supplementary material for: Cloning and endogenous expression of a Eucalyptus grandis UDP-glucose dehydrogenase cDNA
Source: Genet Mol Biol. 2010 Dec 1;33(4):686–95. doi: 10.1590/S1415-47572010005000078 (PMC3036151; doi:10.1590/S1415-47572010005000078)
Supplement: Table S2 — Peptide sequencing data of UGDH proteins shown in Figure 7, identified by LC-MS/MS. [file gmb-33-4-686-suppl2.pdf]

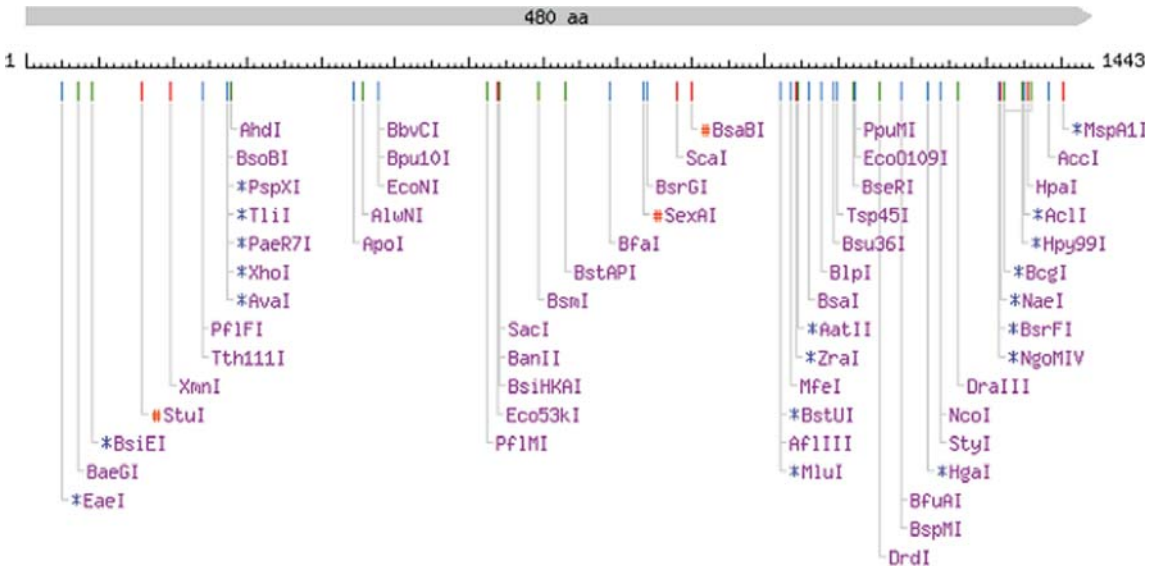

Figure S2 - Restriction map of the cloned *E. grandis UGDH* cDNA.
